# The Synthesis and Biological Evaluation of a Novel Pleuromutilin Derivative Containing a 4-Fluorophenyl Group Targeting MRSA

**DOI:** 10.3390/molecules30112366

**Published:** 2025-05-29

**Authors:** Yongfei Wang, Yi Zhao, Haiting Wang, Bo Liu, Shuangyi Zhang, Yuan Liu, Ruinan Li, Tao Zhang, Surong Hasi, Wei Mao

**Affiliations:** 1 Laboratory of Veterinary Clinical Pharmacology, College of Veterinary Medicine, Inner Mongolia Agricultural University, No. 29, Erdosdong Road, Hohhot 010011, China; 2 Experimental Animal Center, Inner Mongolia Medical University, Hohhot 010110, China; 3 Shandong Provincial Animal and Poultry Green Health Products Creation Engineering Laboratory, Institute of Poultry Science, Shandong Academy of Agricultural Science, Jinan 250100, China

**Keywords:** pleuromutilin derivative, methicillin-resistant *Staphylococcus aureus*, antibacterial activity, molecular docking

## Abstract

The pleuromutilin derivative, the compound PL-W, was synthesized by introducing a 4-fluorophenyl group at the C21 position and selected for comprehensive antibacterial evaluation. PL-W demonstrated notable antibacterial activity against methicillin-resistant *Staphylococcus aureus* (MRSA), with a minimum inhibitory concentration (MIC) of 0.03125 µg/mL, which is significantly lower than that of tiamulin (0.5 µg/mL). Crystal violet (CV) staining revealed that it inhibited MRSA biofilm formation and electron microscopy revealed that it disrupted bacterial cell division and, possibly, the synthesis of essential cell wall proteins. In both in vivo models, PL-W exhibited excellent performance. In the *Galleria mellonella* infection model, treatment with different concentrations of PL-W increased the survival rate from 20% to 90% and significantly reduced the bacterial load. In the mouse model of MRSA pneumonia, a 10 mg/kg dose of PL-W increased the survival rate to 70%, decreased the bacterial load in the lungs, and alleviated inflammatory damage. Molecular docking studies indicated that PL-W had a similar docking pose and comparable binding affinity to that of lefamulin, with hydrogen bond interactions that are crucial for binding to the peptidyl transferase center (PTC). Moreover, it demonstrated no significant reduction in cell viability in HepG2 and HEK293 cells, even at high concentrations (≤50 µg/mL). Overall, PL-W shows significant potential as a novel anti-MRSA agent owing to its potent in vitro and in vivo activities and low cytotoxicity.

## 1. Introduction

Owing to the abuse of antibiotics, drug-resistant bacteria are becoming more common worldwide, posing a significant threat to human health. Methicillin-resistant *Staphylococcus aureus* (MRSA) is a drug-resistant strain of *S. aureus* that shows a high degree of resistance to traditional antibiotics, such as methicillin. This drug resistance complicates the treatment of MRSA infections, particularly in hospital settings [1]. MRSA can cause a variety of infections, including pneumonia and bloodstream, skin, and wound infections. These infections are often challenging to manage and can result in fatalities. Notably, MRSA strains are among the most prevalent pathogens in clinical settings and contribute significantly to global mortality and morbidity. Some researchers predict that approximately 10 million individuals may succumb annually to antibiotic-resistant infections by 2050, surpassing the combined death toll from cancer and diabetes [2]. In recent years, methicillin-resistant MRSA has emerged as a leading cause of mortality associated with drug-resistant bacteria, posing a significant challenge to the prevention and control of global infectious diseases [3]. Therefore, the development of novel antibacterial agents is urgently required [4,5].

**Figure 1:**
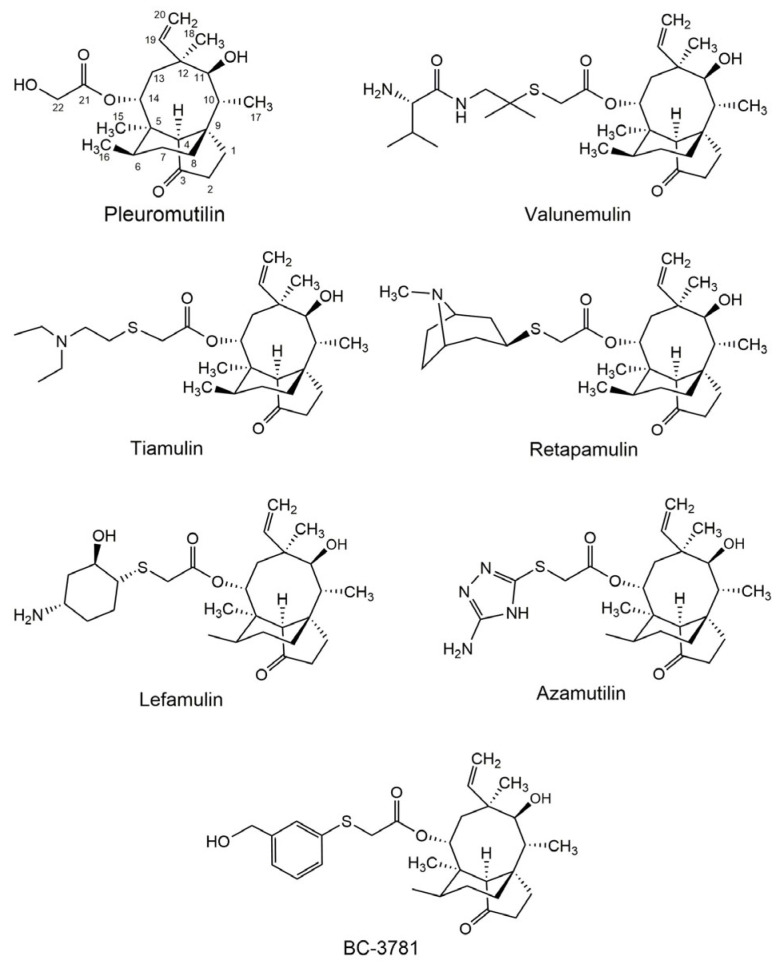
Structures of pleuromutilin, tiamulin, vanemulin, azamutilin, retapamulin, Lefamulin, and BC-7013.

Pleuromutilin is a tricyclic diterpenoid that exhibits potent activity against Gram-positive pathogens, including *S. aureus*, and has a distinct mode of action. It was first isolated from the fungi *Pleurotus mutilus* and *Pleurotus passeckerianus* in the early 1950s. Pleuromutilin works by binding to the 50S subunit of prokaryotic ribosomes, specifically to domain V of 23S rRNA at the peptidyl transferase center (PTC), which inhibits the peptidyl transferase reaction and thus blocks bacterial protein synthesis, but it does not affect eukaryotic protein synthesis. Numerous semisynthetic pleuromutilin derivatives have been designed (Figure 1), synthesized, and evaluated for their antibacterial efficacy [6,7,8,9,10]. Tiamulin and vanemulin have been developed as veterinary drugs by introducing substituents containing sulfide ether bonds at the hydroxyl group of the C14 glycolate ester side chain [11]. At a later stage, the first pleuromutilin derivative for human use, retapamulin, was approved as an antibacterial agent, specifically for treating skin infections [12]. BC-7013 (Figure 1) is a novel pleuromutilin derivative currently used in clinical trials alongside BC-3781 and BC-3205. The use of pleuromutilin in systemic treatments is increasing [13]. The derivatives of pleuromutilin have demonstrated promising efficacy in treating MRSA [8,14,15], a globally recognized challenge, as evidenced by numerous studies.

Fluorine-containing antibacterial agents possess distinct advantages for drug design [16]. The introduction of fluorine atoms can effectively modulate the lipophilicity of a molecule via their strong electronegativity and small atomic radius, thereby enhancing hydrophobic interactions with the target protein [17]. Studies have demonstrated that fluorine substitution can substantially enhance the metabolic stability of compounds while extending their antibacterial activity [18]. We propose a precise fluorination strategy for pleuromutilin, which offers a novel approach for the development of highly efficient and low-toxicity antibacterial agents.

In this study, we designed and synthesized a novel pleuromutilin derivative by introducing a 4-fluorophenyl group at the C21 position to explore its potential applications in veterinary medicine. We systematically evaluated the in vitro and in vivo antibacterial activity of this derivative against MRSA. We also investigated its binding mode to the PTC to clarify its mechanism of action. This study aimed to identify a novel anti-MRSA drug candidate with high antibacterial activity and a favorable safety profile. The discovery of this derivative is expected to enrich current treatment options for MRSA infections, reduce the impact of drug-resistant bacteria, and improve clinical outcomes.

## 2. Results

### 2.1. Synthesis Chemistry

**Scheme 1:**
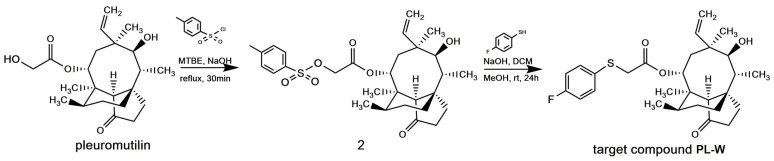
Synthesis route of PL-W.

The synthetic reaction of the pleuromutilin derivatives is shown in Scheme 1. Synthesis of target compound PL-W via nucleophilic substitution pleuromutilin was used as the starting material. Under strongly basic conditions, *p*-toluenesufonyl chloride was employed to activate the hydroxyl group at the C21 position of pleuromutilin. Following the reaction, the mixture was filtered to obtain a white solid (intermediate **2**) [6]. The solid was then washed with methyl tert-butyl ether, dried, and directly used in the subsequent step. In the next stage, 4-fluorobenzenethiol was used for nucleophilic substitution at the activated C21 position. The progress of the reaction was monitored using thin-layer chromatography (TLC). After the completion of the reaction, the product was purified by silica gel column chromatography, yielding a 69% overall recovery. The structures of intermediate **2** and target product PL-W were characterized and confirmed by NMR spectroscopy and HRMS. The purity of intermediate **2** and target product PL-W was characterized and confirmed by HPLC.

### 2.2. In Vitro Antibacterial Activity

#### 2.2.1. Minimum Inhibitory Concentration/Minimum Bactericidal Concentration

The in vitro antibacterial activity of PL-W against common pathogenic bacteria was evaluated with tiamulin as a control. The minimum inhibitory concentrations (MICs) and minimum bactericidal concentrations (MBCs) of the drug against *S. aureus*, MRSA, MRSE, *S. pneumoniae*, *E. coli*, *B. subtilis*, *P. aeruginosa*, and seven clinical isolates of drug-resistant *S. aureus* were determined. The results are presented in Table 1. The MIC of PL-W against *S. aureus* ranged from 0.03125 to 0.25 µg/mL, indicating that lower MIC values were correlated with stronger antibacterial activity. Although a trend of bacteriostatic activity against *S. pneumoniae* MRSE was observed, the antibacterial activity of the compound against these bacteria was low compared to its activity against MRSA. The MIC of MRSA (ATCC33591) was 0.03125 µg/mL, which was significantly lower than that of the control. These results indicate that PL-W exhibits potent activity against MRSA. A time–kill curve analysis was conducted to further investigate the antibacterial dynamics and bactericidal characteristics of PL-W against MRSA.

#### 2.2.2. Time–Kill Kinetics

**Figure 2:**
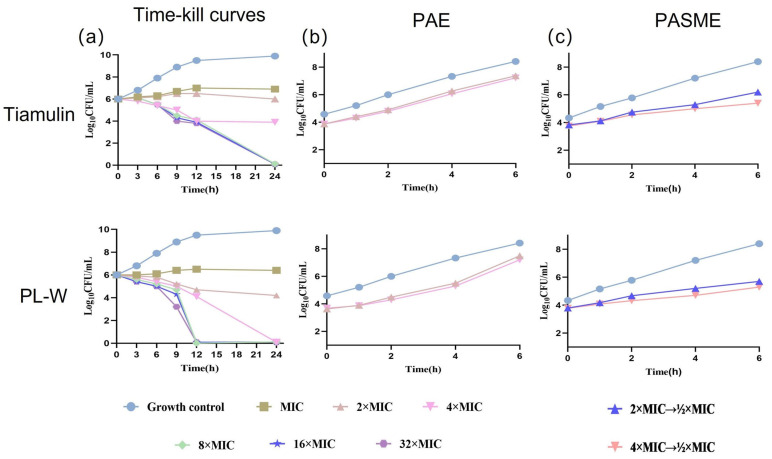
Antibacterial activity and post-antibiotic effects (PAEs) of tiamulin or S-(4-fluorophenyl)-pleuromutilin against MRSA (ATCC33591). (**a**) Time–kill curves for MRSA ATCC33591 with S-(4-fluorophenyl)-pleuromutilin and tiamulin. (**b**) Bacterial growth kinetics after exposure to S-(4-fluorophenyl)-pleuromutilin and tiamulin for 2 h at 4 × and 2 × MIC. (**c**) Post-antibiotic sub-MIC growth curves (PASME). Graphs illustrate the growth of bacteria (expressed as Log_10_CFU/mL) over time for S-(4-fluorophenyl)-pleuromutilin and tiamulin. The bacteria were first treated with 2 × and 4 × MIC concentrations of the respective drugs, after which, the antibiotics were removed, and the concentrations were adjusted to 1/2 × MIC. Note: The MIC of ATCC33591 against S-(4-fluorophenyl)-pleuromutilin was 0.03125 µg/mL and the MIC of ATCC33591 against tiamulin was 0.5 µg/mL.

Using tiamulin as the reference drug, the bactericidal time course of PL-W against MRSA was evaluated (Figure 2a). PL-W showed a significant bactericidal effect, achieving a 99.9% reduction in MRSA at concentrations ≥ 4-fold the MIC. In comparison, tiamulin required concentrations ≥ 8-fold the MIC to attain comparable levels of bactericidal activity. Further studies showed that when the concentration of PL-W reached a certain threshold, the bactericidal efficacy did not increase proportionally with higher concentrations. This indicates that PL-W exhibits time-dependent antibacterial activity. Notably, PL-W achieved a 99.9% reduction in the bacterial count after incubation at 8–32-fold the MIC for 12 h, whereas tiamulin required 24 h of incubation at the same concentration range to achieve a similar effect. These findings indicate that PL-W exhibits a more rapid and potent bactericidal action than tiamulin.

#### 2.2.3. Post-Antibiotic Effect (PAE) and Post-Antibiotic Sub-MIC Effect

The PAE values for PL-W at concentrations 2- and 4-fold the MIC were 0.63 and 0.91 h, respectively, whereas those of tiamulin at the same concentrations were 0.31 and 0.43 h, respectively (Figure 2b, Table 2). The post-antibiotic sub-MIC effect (PASME) values for PL-W at concentrations of 2- and 4-fold the MIC were 1.45 and 2.66 h, respectively, whereas those for tiamulin at the same concentrations were 1.15 and 1.85 h, respectively (Figure 2c, Table 2). This indicates that PL-W exhibits a prolonged inhibitory effect on bacterial growth compared with tiamulin at sub-MIC levels, indicating that PL-W exhibits a significantly longer PAE duration than tiamulin and a more sustained inhibitory effect on bacterial growth, thus potentially permitting extended dosing intervals.

#### 2.2.4. Scanning Electron Microscopy (SEM)

**Figure 3:**
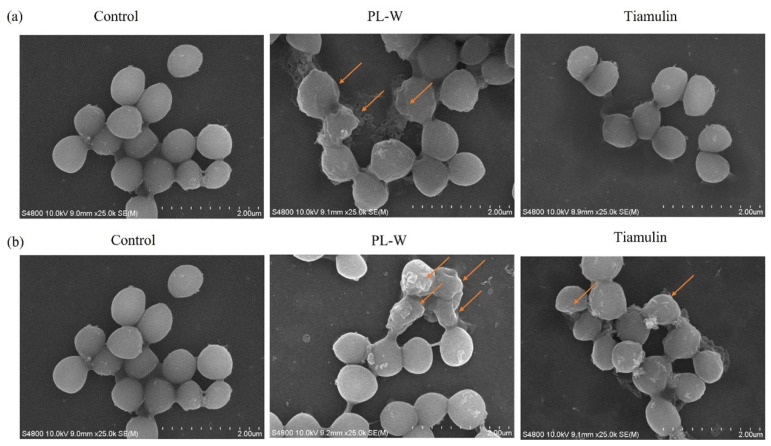
Bacterial morphologies with PL-W and tiamulin treatment groups at (**a**) MIC and (**b**) 4 × MIC. Note: Orange arrows denote damaged sites of bacteria.

To investigate the mechanism of action of PL-W against MRSA, MRSA samples treated with varying concentrations of PL-W were analyzed using SEM. Specifically, the ultrastructural changes in MRSA treated with different concentrations of PL-W and tiamulin were examined. The cell surface of the control group exhibited regular, smooth, and intact morphology encapsulated by a biofilm (Figure 3). At the MIC, PL-W and tiamulin induced varying degrees of surface disruption and suspended division. SEM revealed that at 4-fold the MIC, both PL-W- and tiamulin-treated cells exhibited indistinct biofilms, defects in the cell walls, highly disrupted and distorted morphologies, and significantly compromised structural integrity. Under SEM, no significant difference was observed in the antibacterial efficacy of PL-W against MRSA between the MIC and 4 × MIC. Notably, microscopic analysis revealed that PL-W exhibited superior inhibitory effects on MRSA compared with tiamulin across varying concentrations

#### 2.2.5. Biofilm Inhibition Assay

**Figure 4:**
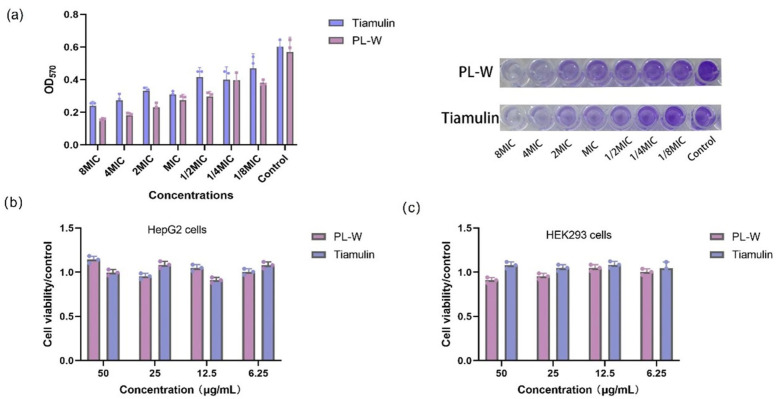
(**a**) Inhibitory effects of different PL-W and tiamulin concentrations on MRSA (ATCC33591) biofilm formation. The effect of PL-W or tiamulin on the viability of HepG2 (**b**) and HEK293 (**c**) cells was assessed using the CCK-8 assay. Note: The MIC of ATCC33591 against PL-W was 0.03125 µg/mL and the MIC of ATCC33591 against tiamulin was 0.5 µg/mL.

Using an electron microscope, we observed the effects of PL-W on bacterial cells and the potential impacts on biofilm formation from 1/8 to 8 × MIC using a CV assay. This study illustrates the impact of PL-W concentration on MRSA biofilm formation. The biofilm inhibition rates at the MIC were 51.40% for PL-W and 48.30% for tiamulin. At 2 ×, 4 ×, and 8 × the MIC, the inhibition rates of PL-W on biofilm formation were 59.67%, 68.13%, and 72.00%, respectively (Figure 4a).

### 2.3. Cell Viability Assay

PL-W exhibited significant anti-MRSA activity. The effects on cellular activity needed to be explored to comprehensively assess its medicinal potential.

The impact of PL-W on cell viability was evaluated in HepG2 (human hepatoma cells) and HEK293 (human embryonic kidney cells) using the CCK-8 assay. These cell lines were selected because (1) the liver and kidneys are central to drug metabolism and excretion, respectively; (2) HepG2 cells retain hepatic metabolic functions and are widely used for detecting drug-induced mitochondrial dysfunction and oxidative stress [19]; and (3) HEK293 cells express renal transporters and serve as robust models for nephrotoxicity screening [10,20,21]. Serial dilutions by a factor of 2 were performed, starting from a maximum concentration of 50 µg/mL for six consecutive steps. No significant effect on cell viability was observed after treating the HepG2 (Figure 4b) and HEK293 (Figure 4c) cells with the tested concentrations of PL-W. In addition, tiamulin exhibited comparable effects on cell viability in both cell lines.

### 2.4. In Vivo Experiment

#### 2.4.1. *G. mellonella*

**Figure 5:**
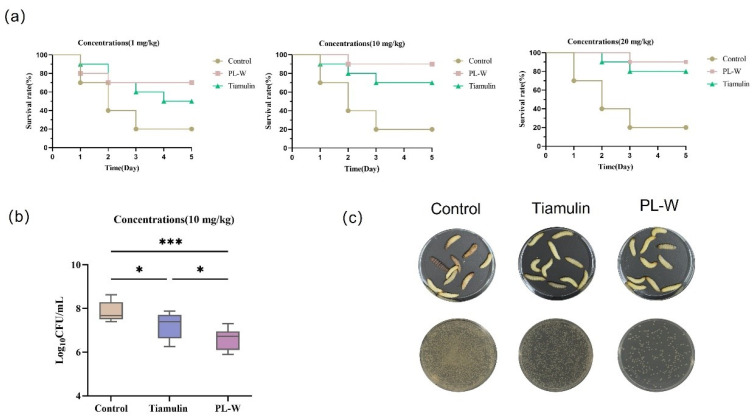
In vivo evaluation of MRSA ATCC33591 infection using the *G. mellonella* larvae model. (**a**) Survival of *G. mellonella* when infected with PL-W or tiamulin-resistant MRSA ATCC33591 (*n* = 10). (**b**) Effects of PL-W and tiamulin in *G. mellonella* larvae 2 h after MRSA ATCC33591 infection. After inoculation with MRSA ATCC33591, larvae were administered PL-W or tiamulin at 10 mg/kg. Number of CFUs in larvae treated with PL-W or tiamulin 8 h post-infection. * *p* < 0.05, *** *p* < 0.001. (**c**) After treatment with PL-W or tiamulin for 8 h, the state of the *G. mellonella* larvae and the bacterial count on the plate diluted 10,000-fold were evaluated.

*G. mellonella* larvae infected with MRSA ATCC33591 showed that the survival rate of the control group was 20%. In contrast, at a concentration of 1 mg/kg, the survival rates of larvae treated with PL-W and tiamulin were 70% and 50%, respectively. Specifically, antibacterial treatment with PL-W increased the survival rate by 50% compared with the control group (Figure 5a). When the drug concentration was increased to 10 mg/kg, the survival rates of the larvae treated with PL-W and tiamulin increased to 90% and 70%, respectively. With further increases to 20 mg/kg, the survival rates of larvae treated with PL-W and tiamulin reached 90% and 80%, respectively. Notably, the survival rate of the PL-W group did not significantly change from 10 to 20 mg/kg; however, the survival time was prolonged from day 2 to 3. The number of culture-forming units (CFUs) recovered from *G. mellonella* larvae infected with MRSA ATCC33591 is shown in Figure 5b,c. At 10 h post-infection, both PL-W and tiamulin treatments resulted in a statistically significant reduction in MRSA CFUs compared with the untreated control group (*p* < 0.001 and *p* < 0.05, respectively). The PL-W treatment group exhibited significantly lower values than the tiamulin treatment group (*p* < 0.05). After verifying the efficacy of PL-W using the greater wax moth larval model, which demonstrated significant therapeutic effects, drug performance was evaluated in a mammalian model to better approximate human physiological conditions.

#### 2.4.2. MRSA-Induced Acute Pneumonia Model in Mice

**Figure 6:**
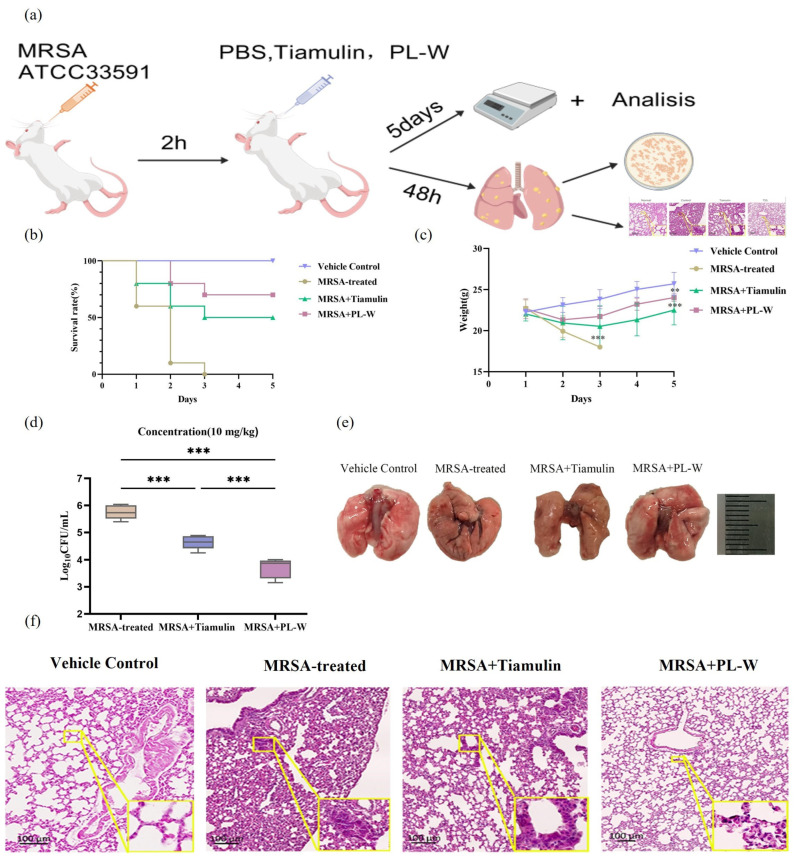
Efficacy and pathological changes in MRSA-induced acute pneumonia model in mice treated with PL-W and tiamulin. Vehicle control: Mice received PBS via pulmonary administration. MRSA-treated: Mice were inoculated with MRSA to establish infection, with no subsequent therapeutic intervention. MRSA + tiamulin/PL-W: MRSA-infected mice were treated with tiamulin/PL-W. (**a**) Verification flowchart for MRSA-induced acute pneumonia model in mice treated with PL-W and tiamulin. (**b**) Temporal changes in the survival rates of mice within each experimental group are indicated. (**c**) Weight change curves of mice in the different groups. Compared with the vehicle control, ** *p* < 0.01, and *** *p* < 0.001. (**d**) Comparison of Log_10_CFU/mL in lung tissues among MRSA, tiamulin (10 mg/kg), and PL-W (10 mg/kg) groups. (**e**) Apparent map of lung in each group. (**f**) Histopathological sections of lung tissues from each group of mice were stained with H&E. The yellow box indicates the locally magnified area for observing pathological changes in lung tissue structure.

Mice were infected with MRSA ATCC33591 to establish an acute pneumonia model. The survival rate in the vehicle control group was 100%, whereas that in the MRSA-treated group was 0% by day 3. It is essential to highlight that at an administration concentration of 10 mg/kg, the survival rate for PL-W was 70%, whereas the survival rate for tiamulin was 50%. The body weight of mice in the vehicle control group remained stable, whereas that of mice in the MRSA-treated group decreased until death. In the tiamulin-treated and PL-W-treated groups, body weight decreased during the first 2 days but recovered thereafter. Compared with the vehicle control group, the weight of mice in the MRSA + PL-W group was different (*p* < 0.01), whereas the weight of mice in the MRSA and MRSA + tiamulin groups was significantly different (*p* < 0.001). The plate count results for lung tissue showed that the number of colonies in the MRSA + PL-W group was significantly lower than that in the MRSA-treated group and the MRSA + tiamulin group *(p* < 0.001). In terms of organ appearance, the lung tissue in the vehicle control group exhibited uniform color and luster, was soft and elastic in texture, and showed no evident lesions. In contrast, the MRSA-treated group exhibited a markedly deeper dark red color with a rough and uneven surface. The MRSA + tiamulin and MRSA + PL-W groups significantly improved. Hematoxylin and eosin (H&E)-stained sections demonstrated that the lung tissue structure in the vehicle control group was well preserved. In contrast, the MRSA-treated group exhibited extensive inflammatory foci with a significant aggregation of inflammatory cells within the alveolar structures. Both the tiamulin-treated and PL-W-treated groups showed improvements compared to the MRSA-treated group. The MRSA + PL-W group exhibited a clearer alveolar tissue structure with reduced inflammatory cell infiltration in the alveolar spaces, indicating superior repair and the lowest degree of injury. Collectively, PL-W exhibited potent antibacterial effects in vivo in our mouse model (Figure 6).

### 2.5. Molecular Docking Study

**Figure 7:**
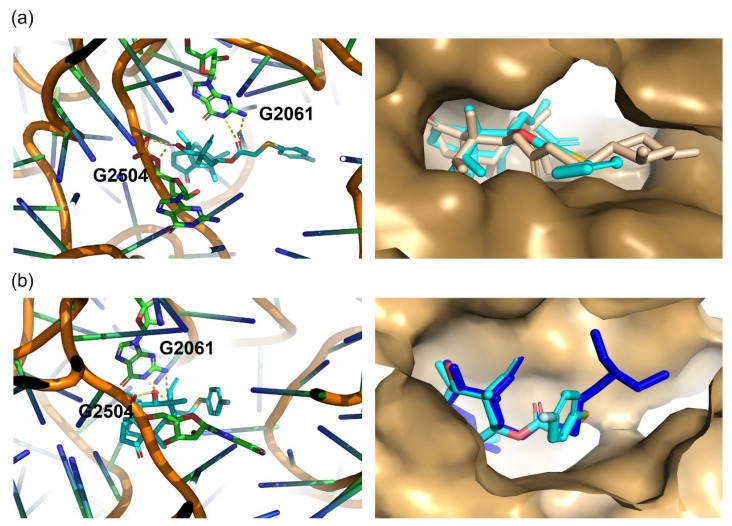
(**a**) The simulated docking modes of PL-W (cyan) and lefamulin (wheat) to 5HL7. (**b**) The simulated docking modes of PL-W (cyan) and tiamulin (blue) to 1XBP. G2061 and G2504 residues are represented in green.

Molecular docking was performed using the *S. aureus* ribosome (PDB ID: 5HL7) [22] as the receptor (Figure 7) with Smina software [23], which is based on AutoDock Vina (V.1.2.0) [24,25] but incorporates improvements in the scoring function through data training. These enhancements allow Smina to better reflect the interaction patterns between the ligand and ribosome. To validate our docking procedure, we first redocked lefamulin and achieved a root mean square deviation (RMSD) of 0.71. This indicated that our docking results accurately reflected the binding mode between the ligand and receptor. The docking results revealed that the affinity of the compound PL-W to the ribosome (PDB ID: 5HL7) was −9.7 kcal/mol, whereas the affinity of lefamulin was −9.5 kcal/mol (Supplementary Table S3). This indicates that PL-W shares a similar docking pose with lefamulin and has a comparable binding affinity. In contrast, the docking results of tiamulin with 5HL7 showed a binding energy of only −8.7 kcal/mol, further explaining why PL-W exhibits better antibacterial activity. The interaction of PL-W with the ribosomal residues G2061 and G2504 (numbered based on the *Escherichia coli* ribosome) through hydrogen bonds is a key factor in its binding to the PTC. In addition, van der Waals interactions with other residues significantly contributed to the inhibitory effect of PL-W on bacterial protein synthesis. We performed molecular docking of PL-W to the ribosomal structure of 1XBP and analyzed it. Analysis revealed that the PL-W at 5HL7 and 1XBP docking results showed that they highly overlapped with the structural mutilin ring of the original ligand and formed hydrogen bonds with the same number of bases.

## 3. Experimental Section

### 3.1. Materials and Chemicals

Cells were obtained from the Stem Cell Bank of the Chinese Academy of Sciences (Shanghai, China). The *S. aureus* strains ATCC29213 and ATCC33591 were obtained from the American Type Culture Collection (ATCC; Manassas, VA, USA). Experimental mice were provided by Inner Mongolia Medical University, China (license number SCXK [Inner Mongolia] 2020-0001), 2020–2025. All animal experiments were approved by the Experimental Animal Welfare and Ethics Committee of Inner Mongolia Agricultural University, and all animal procedures adhered strictly to the guidelines and regulations established by the University Animal Protection Committee. Unless otherwise specified, all chemicals were purchased from Sigma-Aldrich (St. Louis, MO, USA). ^1^H NMR and ^13^C NMR data were collected using the Bruker AVANCE III HD 500 instrument (Zug, Switzerland). The compound was purified using column chromatography with 200–300 mesh silica gel. Mass spectrometry was performed using the Thermo Q Exactive Plus (Waltham, MA, USA) equipped with electrospray ionization (ESI) sources. Purity analysis was performed using a Thermo U3000 (Waltham, MA, USA) and Agilent 1260 HPLC (Santa Clara, CA, USA) system equipped with a UV detector.

### 3.2. Synthesis of Intermediate **2** and S-(4-fluorophenyl)-pleuromutilin

Pleuromutilin (3.78 g, 1.0 equiv) was dissolved in methyl tert-butyl ether (MTBE, 30 mL). *p*-toluenesulfonyl chloride (2.27 g, 1.2 equiv) was added to the solution. Aqueous NaOH (10 N, 2.0 equiv, 2 mL) was added dropwise and the mixture was refluxed for 1 h. The reaction mixture was filtered to collect the residue, which was washed with MTBE and dried under a vacuum, yielding intermediate **2** that was characterized and soon used for the consecutive reaction, without any further purification. Intermediate **2** (0.53 g, considered to be 1.0 equiv) was dissolved in dichloromethane (DCM, 10 mL). 4-Fluorothiophenol (0.24 g, 1.0 equiv) was added to the solution. A methanolic NaOH solution (5 wt%, 1.2 equiv, 1 mL) was added dropwise and the mixture was stirred at room temperature for 24 h. The reaction mixture was concentrated under reduced pressure and the residue was dissolved in DCM (20 mL). The organic layer was extracted with saturated NaCl solution (10 mL × 2). The combined organic phases were dried over anhydrous Na_2_SO_4_, filtered, and concentrated. The crude product was purified by silica gel column chromatography using a mobile phase of petroleum ether (PE): ethyl acetate (EA) = 1:1. A pale yellow solid was obtained (yield: 69%, 0.198 g). The target product, S-(4-fluorophenyl)-pleuromutilin (PL-W), was characterized and confirmed using nuclear magnetic resonance (NMR) spectroscopy.

Intermediate **2**. ^1^H NMR (500 MHz, DMSO-*d*6) δ 7.81 (d, *J* = 7.9 Hz, 2H), 7.48 (d, *J* = 7.9 Hz, 2H), 6.20–5.95 (m, 1H), 5.54 (d, *J* = 8.4 Hz, 1H), 5.07–4.99 (m, 2H), 4.73 (ddd, *J* = 60.7, 19.6, 10.9 Hz, 2H), 4.60–4.48 (m, 1H), 3.41 (t, *J* = 6.1 Hz, 1H), 2.42 (s, 3H), 2.40 (s, 1H), 2.17 (d, *J* = 11.0 Hz, 1H), 2.11 (d, *J* = 19.4 Hz, 1H), 2.01 (s, 2H), 1.63 (s, 2H), 1.46 (s, 1H), 1.38 (d, *J* = 6.4 Hz, 1H), 1.32 (s, 3H), 1.28 (s, 2H), 1.26 (s, 1H), 1.09–1.02 (m, 3H), 1.00 (s, 1H), 0.82 (d, *J* = 6.9 Hz, 3H), 0.52 (t, *J* = 10.6 Hz, 3H). ^13^C NMR (125 MHz, DMSO-*d*6) δ 217.5 (C), 165.2 (C), 145.7 (C), 141.1 (C), 132.7 (C), 130.6 (2 × CH), 128.2 (2 × CH), 115.8 (CH), 73.0 (CH), 70.8 (CH), 66.2 (CH), 57.6 (CH), 45.4 (C), 44.6 (CH_2_), 43.6 (CH_2_), 42.0 (C), 37.0 (CH_2_), 36.7 (CH), 34.4 (CH_2_), 30.5 (CH_2_), 29.1 (CH_2_), 27.0 (CH_3_), 24.9 (CH_2_), 21.6 (CH_3_), 16.3 (CH_3_), 14.9 (CH_3_), 12.0 (CH_3_). (Supplementary Figure S1). HRMS(ES) calculated [M + H]^+^ for [C_29_H_41_O_7_S]^+^ 533.2573, found to be 533.2574 (Supplementary Figure S2). The purity of intermediate **2** was 98.1858% by HPLC (Supplementary Figure S3 and Table S1).

A pale yellow solid was obtained (yield: 69%). ^1^H NMR (500 MHz, DMSO-*d*6) δ 7.72–7.36 (m, 2H), 7.17 (dd, *J* = 8.7, 2.0 Hz, 2H), 6.04 (d, *J* = 6.3 Hz, 1H), 5.49 (d, *J* = 8.2 Hz, 1H), 5.06–4.85 (m, 2H), 4.52 (d, *J* = 5.9 Hz, 1H), 3.94–3.71 (m, 2H), 3.39 (s, 1H), 2.37 (s, 1H), 2.16 (d, *J* = 10.8 Hz, 1H), 2.10–2.04 (m, 1H), 2.04–1.90 (m, 2H), 1.71–1.54 (m, 2H), 1.44 (d, *J* = 9.0 Hz, 1H), 1.41–1.34 (m, 1H), 1.30 (d, *J* = 1.8 Hz, 3H), 1.28–1.21 (m, 2H), 1.21–1.10 (m, 1H), 1.03 (d, *J* = 17.6 Hz, 1H), 1.00 (s, 3H), 0.80 (d, *J* = 7.0 Hz, 3H), 0.55 (d, *J* = 6.9 Hz, 3H). ^13^C NMR (126 MHz, DMSO-*d*6) δ 217.6 (C), 168.0 (C), 162.5 (CF), 160.6 (CF), 141.2 (2 × CH), 132.0 (CH), 131.9 (CH), 130.8 (C), 116.5 (CH_2_), 116.3 (CH), 115.6 (CH), 73.0 (CH), 70.2 (CH), 57.7 (CH), 45.4 (C), 44.4 (CH_2_), 41.9 (C), 36.8 (CH_2_), 36.6 (CH), 34.4 (CH_2_), 30.6 (CH_2_), 29.0 (CH_2_), 27.0 (CH_3_), 25.0 (CH_2_), 16.5 (CH_3_), 14.9 (CH_3_), 12.0 (CH_3_). ^19^F NMR (471 MHz, DMSO-*d*6) δ -115.77 (m) (Supplementary Figure S4). HRMS(ES) calculated [M + Na]^+^ for [C_28_H_37_FO_4_SNa]^+^ 511.2284, found to be 511.22842 (Supplementary Figure S5). The purity of S-(4-fluorophenyl)-pleuromutilin (PL-W) was 96.003% by HPLC (Supplementary Figure S6 and Table S2).

### 3.3. In Vitro Efficacy of PL-W

#### 3.3.1. MIC/MBC Testing

The synthesized PL-W was dissolved in dimethyl sulfoxide (DMSO) to prepare a stock solution (128 mg/mL). PL-W or tiamulin was serially diluted two-fold in PBS in a 96-well plate, resulting in concentrations of 0.977 ng/mL to 2 µg/mL. For some strains, the concentration range of PL-W was expanded to 64 µg/mL by dilution with a stock solution of 512 µg/mL. Standard strains of MRSA (ATCC33591), MRSE (ATCC51625), *S. aureus* (ATCC29213), *S. pneumoniae* (ATCC49619), *E. coli* (ATCC25922), *B. subtilis* (ATCC21216), *P. aeruginosa* (ATCC14215), and seven clinical isolates of MRSA (derived from drug-resistant *Staphylococcus aureus* isolated from animal clinical samples) were cultured in appropriate media until they reached the logarithmic growth phase. Subsequently, the bacterial suspensions were adjusted to 1 × 10^6^ CFU/mL using the respective medium and inoculated into 96-well plates containing varying concentrations of the test compounds. Each well received 200 µL of inoculum, resulting in a final bacterial density of 5 × 10^5^ CFU/mL. Three parallel experiments were performed, including both positive and negative controls. The 96-well plates were incubated at 37 °C for 18 h to determine the MIC. Subsequently, an aspirate (25 µL) of the bacterial suspension obtained from the wells that showed no visible growth was inoculated onto MH agar plates. The plates were then incubated at 37 °C for 18 h. For isolates with MIC ≥ 64 µg/mL, results were reported as ‘>64 µg/mL’ to reflect resistance thresholds. The colonies on the plates were then counted to determine the MBC value [26].

#### 3.3.2. Time–Kill Curves

Based on previously determined MIC values, PL-W and tiamulin solutions were prepared at concentrations 1–32-fold the MIC in MH broth. Methicillin-resistant MRSA ATCC33591 cultures in the logarithmic growth phase were adjusted to a concentration of 2 × 10^6^ CFU/mL using MH broth as a diluent. The bacterial suspension was then added to disposable shaker tubes containing varying concentrations of the tested compounds, and a control group was established. The tubes were kept semi-capped to permit air exchange and incubated in a constant-temperature shaker at 37 °C and 180 rpm for 24 h. At 0, 3, 6, 9, 12, and 24 h post-incubation, sterile samples were collected for serial dilution (5–10 fold); this was followed by plate counting. The inoculated plates were incubated at 37 °C for 18 h. This experiment was performed in triplicate. Time–kill curves for the bacteria were constructed by plotting Log_10_ values on the y-axis against time on the x-axis [27,28].

#### 3.3.3. PAE and PA-SME Assays

MRSA ATCC33591 in the logarithmic growth phase was diluted to 10^6^ CFU/mL in MH broth. Aliquots of this bacterial suspension were transferred into separate test tubes, and PL-W was added to achieve final concentrations of 2 × MIC or 4× MIC. A control group containing only a bacterial suspension without PL-W was also established. All samples were incubated in a shaking incubator at 37 °C for 2 h. The MH broth was then serially diluted 1000-fold and the treated bacterial suspensions were incubated at 37 °C. Colony counts were performed on the plates after serial dilutions (ranging from 5 to 1000-fold) at 0, 1, 2, 4, and 6 h post-drug removal [10]. The PAE was calculated using the following formula:

PAE = TA − TC.(1)

The post-antibiotic effects of PL-W on MRSA were evaluated by comparing the results across different experimental groups relative to the control group.

PA-SME was processed as described above for a 2 h incubation period. The supernatant was discarded after centrifugation and the bacterial suspension was resuspended in MH broth. PL-W was added to each diluted bacterial suspension, except for the control group, to achieve a final concentration of 1/2 × MIC. Bacterial suspensions were serially diluted at 0, 1, 2, 4, and 6 h post-treatment and colony-forming units were counted on agar plates. The PA-SME was calculated as follows [29]:

PA-SME = TA − TC.(2)

#### 3.3.4. SEM Assays

The effects of PL-W and tiamulin on MRSA morphology were examined using SEM. ATCC33591 cells were incubated with either PL-W or tiamulin at 37 °C and 180 r/min in a shaker for 12 h. Following incubation, the bacteria were fixed on glass slides using 2 mL of 2.5% glutaraldehyde and stored at 4 °C for 1 h. Subsequently, gradual dehydration was performed using ethanol solutions of increasing concentrations (50%, 70%, 80%, 90%, and 95%) for 20 min each, followed by treatment with pure ethanol for 1 h. The samples were then dried, metalized, and examined using SEM [30].

#### 3.3.5. Biofilm Influence Assays

The concentration of MRSA (ATCC33591) was adjusted to 5 × 10^4^ CFU/mL, and 100 µL of this suspension was inoculated into each well of a 96-well plate. Various concentrations of PL-W (1/8 × MIC–8 × MIC) were added to each well to achieve a final volume of 200 µL/well. The plates were then incubated at 37 °C for 24 h. Following incubation, the wells were washed thrice with phosphate-buffered saline (PBS) and fixed with 99% methanol for 15 min. The methanol was removed and the plate was allowed to dry. The cells were stained with a 0.1% CV solution in the dark for 10 min and then rinsed thrice with PBS to remove excess stain. Next, 95% ethanol was added to each well to dissolve the CV. The absorbance was measured at 570 nm through the optical density (OD) using a microplate reader [31,32] and analyzed using the following formula:

Biofilm inhibition (%) = [OD_570_(control) − OD_570_(sample)]/OD_570_(control) × 100%. (3)

#### 3.3.6. Cell Viability Assay

HepG2 and HEK293 cells [33,34] were selected for further experiments based on the methods used in previous studies. Cells were seeded in a 96-well plate at a density of 1 × 10^5^ cells/well and incubated at 37 °C for 4 h to ensure stable adhesion and growth [35]. Subsequently, the cells were treated with either PL-W or tiamulin at concentrations of 1.5, 3, 6, 12.5, 25, and 50 µg/mL. The treated cells were then incubated at 37 °C under 5% CO_2_ for 24 h to facilitate complete interaction between the compounds and the cells. A cell culture medium containing 10% CCK-8 reagent was added to each well. The plate was incubated at 37 °C for 30 min to facilitate color development. Absorbance was measured at 450 nm using a multifunctional microplate reader and the OD was calculated [36]. Cell viability was calculated using the following formula:

Cell viability = (OD value of the test group − OD value of the cell-free control)/(OD value of the blank control − OD value of the cell-free control).(4)

### 3.4. In Vivo Assays

#### 3.4.1. MRSA Infection Model in *G. mellonella* Larvae and Treatment

*G. mellonella* larvae were selected for the experiments, and the MRSA ATCC33591 concentration was adjusted to 1 × 10^7^ CFU/mL. Fresh antibiotic stock solutions were prepared, which were then diluted to the required concentrations in PBS. Using an insulin syringe, 20 µL of the MRSA suspension was injected into the hemocoel of the larvae through the last pair of left prolegs to induce infection. After infection, the larvae were incubated at 37 °C for 2 h. Subsequently, the drug stock solution was administered into the hemocoel of each larva at concentrations of 1, 10, and 20 mg/kg [37], with an injection volume of 20 µL. PBS was injected into the positive control group. The larvae were then transferred to Petri dishes and incubated at 37 °C for 120 h. During this period, daily observations were conducted to monitor the activity, cocooning behavior, growth, melanization, and survival rate [38].

#### 3.4.2. Determination of *S. aureus* Burden in *G. mellonella* Larvae

The treatment followed the experimental procedure described in Section 3.4.1. Petri dishes were incubated at 37 °C for 8 h. Following incubation, larvae were frozen at −20 °C for 5 min, surface-disinfected with 75% ethanol, and subsequently homogenized in PBS using a tissue grinder. Homogenates were serially diluted between 1:5 and 1:100, and 10 µL aliquots were inoculated onto agar plates. Plates were incubated at 37 °C for 24 h, after which, the CFUs were counted [39].

#### 3.4.3. Mouse Model of MRSA-Induced Acute Pneumonia

A mouse model of acute MRSA pneumonia was established using MRSA ATCC33591 cells. In multiple experimental trials, an infection concentration of 1 × 10^7^ CFU/mL resulted in a mortality rate of >80% in mice. Forty Kunming mice were divided into four groups (*n* = 10 each). The vehicle control group consisted of mice that were not infected with MRSA, whereas the MRSA-treated group comprised mice serving as models for MRSA-induced acute pneumonia without receiving any drug treatment. Mice were anesthetized through respiratory anesthesia, and an MRSA suspension (1 × 10^7^ CFU/mL) was administered intratracheally. The mice were gently shaken to ensure an even distribution of MRSA in both lungs. Two hours post-infection, the MRSA + tiamulin and MRSA + PL-W groups were administered their respective treatments at a dose of 10 mg/kg. Two hours post-infection, the MRSA + tiamulin group and the MRSA + PL-W group were administered a single dose of 10 mg/kg, respectively. Over the subsequent 5-day period, the number of deceased mice was documented, and the mortality rate was calculated accordingly. The mortality rate and body weight of the mice were recorded over the following 5 d. Using the same administration method and dose, another set of vehicle control, MRSA-treated, MRSA + tiamulin, and MRSA + PL-W groups (*n* = 10) were euthanized 48 h post-infection. The lungs were collected, weighed, homogenized, and diluted 10-fold for plate counting. Lung tissue samples were collected for H&E staining.

### 3.5. Molecular Docking

Three-dimensional structures of the ribosomes were obtained from the Protein Data Bank (PDB; PDB IDs: 5HL7 and 1XBP). Water molecules and metal ions were removed from the structures to prevent interference with docking. The structures were then protonated and prepared using AutoDockTools (V.1.2.0) to ensure proper charge states, and non-bonded hydrogen atoms were added. The ligand molecule was constructed, and its energy was minimized using Chem3D 20.1 to optimize its geometry. The minimized structure was saved in an appropriate format for docking analysis. Molecular docking was performed using Smina software. The docking pocket was centered at coordinates (17, −77.9, and −2.6) and the pocket was 20 × 20 × 20 Å. This pocket was selected based on the known ribosome-binding site. The ligand was flexibly docked in a defined pocket. A total of 24 docking conformations were generated, and the binding energy of each conformation was calculated. The docking results were analyzed by ranking the generated conformations based on their binding energies. The most favorable docking pose was selected for further analysis. Interactions between the ligand and ribosome, including hydrogen bonds, hydrophobic interactions, and key amino acid residues involved in binding, were visualized using PyMOL 2.5.

### 3.6. Statistical Analysis

Statistical analyses were conducted using Prism software 9.5 (GraphPad Software, San Diego, CA, USA). One-way and two-way analysis of variance (ANOVA) were used to assess differences among groups. Data are presented as means ± standard deviations (SDs), unless otherwise specified. Statistical significance was set at *p* < 0.05.

## 4. Discussion and Conclusions

In this study, a derivative of the pleuromutilin was tested and showed efficacy against MRSA. We comprehensively evaluated its potential as an MRSA inhibitor, including its synthesis process, antibacterial activity, cytotoxicity, and in vivo antibacterial performance.

Pleuromutilin is known for its significant antibacterial activity against Gram-positive bacteria [9]. Traditional pleuromutilins, such as valnemulin and retapamulin, affect bacterial ribosomes, thereby inhibiting protein synthesis. Through electron microscopy and molecular docking, we determined that PL-W has the same bactericidal mechanism as unmodified pleuromutilin. Although previous studies by Juan Xia et al. have shown that modified pleuromutilin derivatives can disrupt bacterial cell membranes [15], similar investigations have not been conducted for PL-W. Although our study demonstrated that PL-W can inhibit biofilm formation, the mechanism by which it clears pre-existing biofilms remains unclear and requires further investigation.

In terms of antibacterial activity, PL-W demonstrated superior antibacterial and bactericidal efficacy compared with tiamulin against the standard MRSA strain and seven clinical isolates. Notably, PL-W exhibited a 16-fold increase in potency against MRSA ATCC33591. However, its antibacterial activity was less pronounced against other pathogenic bacteria. Consequently, we conducted further investigations into the in vitro and in vivo antibacterial activity of PL-W against MRSA. The in vitro observed PAE and PA-SEM of PL-W provide critical insights into its superior in vivo efficacy. Our data demonstrate that the PAE and PA-SME of PL-W are significantly prolonged compared to tiamulin. These extended inhibitory effects may account for the sustained suppression of bacterial regrowth during the dosing interval, which is consistent with the enhanced survival rates observed in both *Galleria mellonella* and mouse models of pneumonia. This phenomenon is consistent with previous studies linking extended PAE to the improved in vivo efficacy of antibiotics. For instance, Dong et al. demonstrated that pleuromutilin derivatives with longer PAE durations exhibited superior bacterial clearance in *G. mellonella* models, as the prolonged suppression of bacterial regrowth minimized tissue damage and improved host survival [37]. Similarly, Boyer et al. reported that PA-SME plays a pivotal role in maintaining sub-therapeutic drug concentrations to prevent biofilm resurgence in systemic infections [29]. Our findings corroborate these observations, as PL-W-extended PA-SME likely contributed to the significant reduction in lung bacterial load and attenuated inflammatory damage in the murine pneumonia model.

The in vitro cell viability assay demonstrated that PL-W exerted no significant impact on the metabolic activity of HepG2 (human hepatoma cells) and HEK293 (human embryonic kidney cells) at concentrations below 50 µg/mL, indicating its favorable biocompatibility. HepG2 and HEK293 were selected as representative models for two key reasons: liver and kidney involvement in drug metabolism. HepG2 cells retain hepatic CYP450 enzyme activity and bile acid transporters, enabling the assessment of drug-induced mitochondrial dysfunction and oxidative stress [19]. HEK293 cells express renal transporters, mimicking renal tubular secretion and nephrotoxicity mechanisms [20]. However, considering the differences between in vitro and in vivo environments, comprehensive toxicity evaluations and in vivo animal studies are required to clarify its effects on tissues and organs as well as potential adverse reactions.

Molecular docking results revealed that PL-W demonstrates better inhibitory activity against MRSA than tiamulin, with docking energy differences of 1 kcal/mol in 5HL7 and 0.2 kcal/mol in 1XBP, both indicating stronger binding of PL-W compared to tiamulin. In contrast, the mechanistic implications of lefamulin require further investigation, as the subtle differences in binding cannot be fully resolved by static docking alone. Additionally, the solubility of the drugs raises questions; in our experiments, PL-W demonstrated good solubility in DMSO, PBS, and M-H broth. Given its structural similarity to lefamulin, there may be similarities in pharmacokinetics that warrant further study. Future work will involve dynamic simulations and comparative pharmacokinetic analyses to elucidate these differences and provide a comprehensive evaluation of PL-W.

In conclusion, the synthetic process of PL-W is feasible. It exhibits excellent antibacterial activity against MRSA, shows low in vitro cytotoxicity, and has a good therapeutic effect in vivo. These results highlight the significant potential of PL-W as an anti-MRSA drug, which provides a promising option for treating MRSA infections and expands our understanding of combating such infections.

## Figures and Tables

**Table 1:** The MIC and MBC values (µg/mL) of PL-W or tiamulin against MRSA, *S. aureus*, and common pathogenic bacteria were determined.

Test Compounds	MIC/MBC (µg/mL)	
	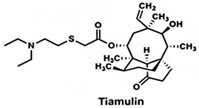	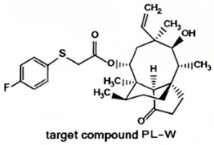
*S. aureus* (ATCC29213)	0.5/1	0.25/1
MRSA (ATCC33591)	0.5/2	0.03125/0.25
MRSE(ATCC51625)	0.5/1	0.125/0.5
SA-R20220105	1/>2	0.125/0.5
SA-R20220106	0.5/2	0.0625/0.5
SA-R20220111	>2/>2	0.125/0.5
SA-R20230119	2/>2	0.25/1
SA-R20240101	1/>2	0.125/0.5
SA-R20240112	1/>2	0.25/0.5
SA-R20240125	1/>2	0.25/2
*S. pneumoniae*ATCC49619	0.5/1	0.125/0.5
*E. coli*ATCC25922	>64	>64
*B. subtilis*ATCC21216	>64	>64
*P. aeruginosa*ATCC14215	>64	>64

**Table 2:** Tabulated PAE and PA-SME durations (determined as the difference in time required for bacterial counts to increase by 10 × between experimental and untreated control arms).

Compounds	Concentrations	PAE (h)	PASME (h)
Tiamulin	2 × MIC	0.31	1.15
	4 × MIC	0.43	1.85
PL-W	2 × MIC	0.63	1.45
	4 × MIC	0.91	2.66

## Data Availability

The original contributions presented in this study are included in the article/Supplementary Materials. Further inquiries can be directed to the corresponding author.

## Supplementary Materials

The following supporting information can be downloaded at https://www.mdpi.com/article/10.3390/molecules30112366/s1: Figure S1: ^1^H NMR and ^13^C NMR spectrum of intermediate **2**; Figure S2: HRMS profile of intermediate **2**; Figure S3: HPLC chromatogram of intermediate **2**; Figure S4: ^1^H NMR, ^13^C NMR and ^19^F NMR spectrum of PL-W; Figure S5: HRMS profile of PL-W; Figure S6: HPLC chromatogram of PL-W; Figure S7: 2D plot of 1XBP; Table S1: HPLC integral table of intermediate **2**; Table S2: HPLC integral table of PL-W; Table S3: Docking scores and intermolecular interactions and distances.
