# Supplementary material for: The Synthesis and Biological Evaluation of a Novel Pleuromutilin Derivative Containing a 4-Fluorophenyl Group Targeting MRSA
Source: Molecules. 2025 May 29;30(11):2366. doi: 10.3390/molecules30112366 (PMC12156078; doi:10.3390/molecules30112366)
Supplement: Supplementary file 1 [file molecules-30-02366-s001.zip › molecules-3606068-supplementary.pdf]

Article

The Synthesis and Biological Evaluation of a Novel Pleuromutilin Derivative Containing a 4-Fluorophenyl Group Targeting MRSA

Yongfei Wang [1,2], Yi Zhao [1], Haiting Wang [3], Bo Liu [1], Shuangyi Zhang [1], Yuan Liu [1], Ruinan Li [1], Tao Zhang [1], Surong Hasi [1,*] and Wei Mao [1,*]

Supplementary material:

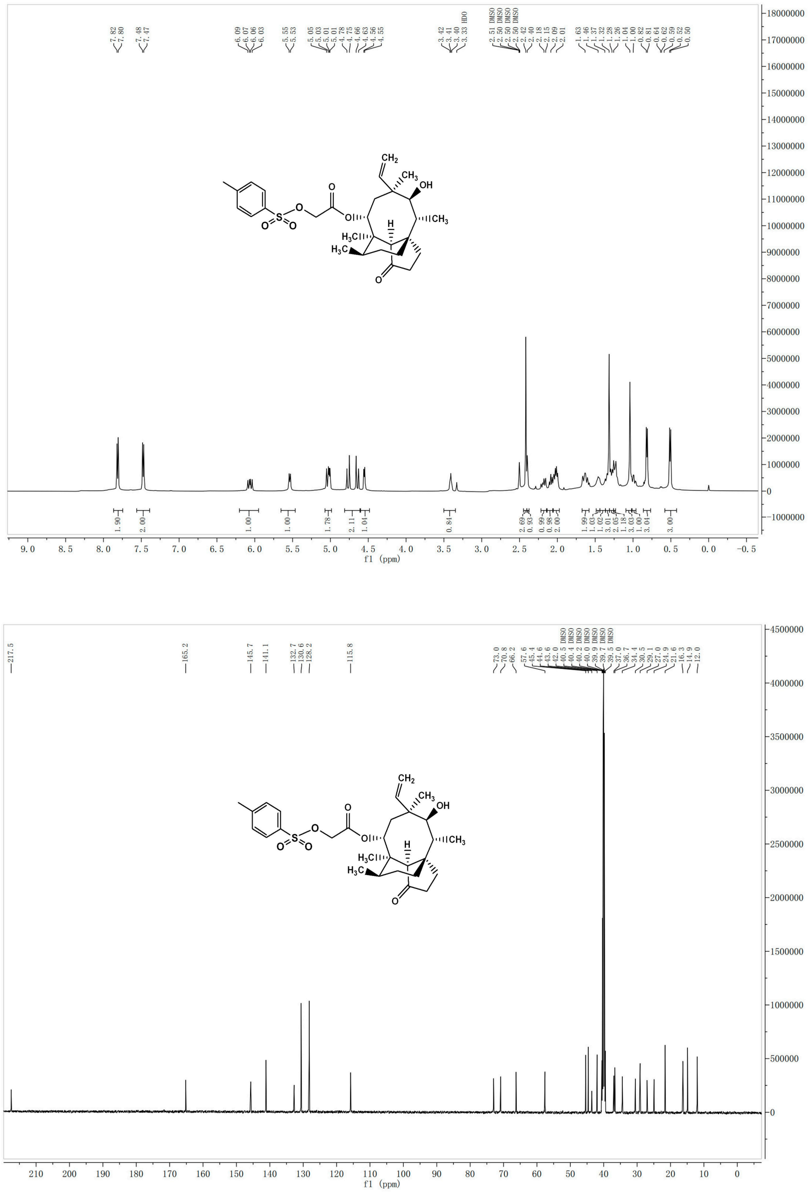

Figure S1. ^{1}H NMR and ^{13}C NMR spectrum of Intermediate **2**.

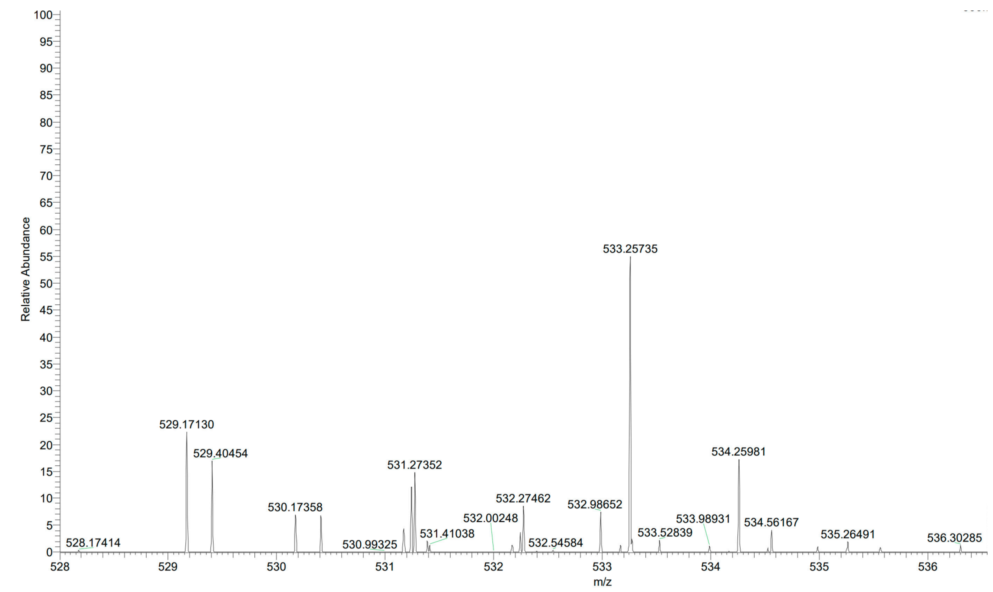

Figure S2. HRMS profile of intermediate **2**.

Method Info: Agilent Zorbax SB-C18 (250 x4.6 mm, 5μm), 1.000ml/min, A: H_2O, B: methanol. Equilibration was carried out at a flow rate of 1 mL/min with a A/B solution (95.0/5.0). The gradient program was set as follows: 0–5 min at 20% B, 5–10 min linear gradient to 80% B, 10–23 min to 90% B and 23–30 min to 5% B.

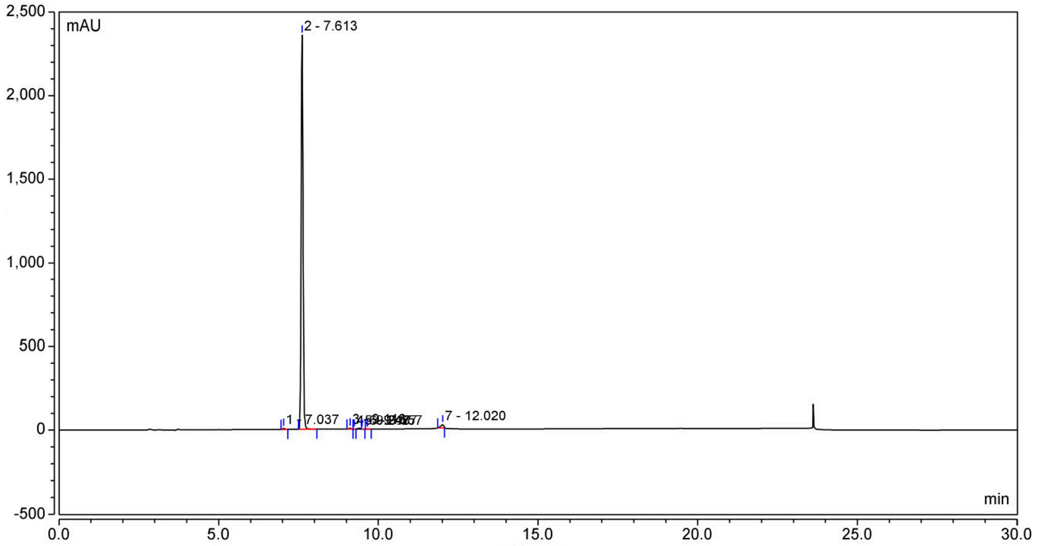

Figure S3. HPLC chromatogram of intermediate **2**.

Table S1. HPLC integral table of intermediate **2**

Peak	RetTime	Area(mAU*min)	Height(mAU)	Area%
1	7.037	0.469	6.414	0.2902
2	7.613	158.571	2358.364	98.1858
3	9.113	0.360	5.928	0.2226
4	9.247	0.011	0.234	0.0069
5	9.487	0.031	0.578	0.0194
6	9.657	0.007	0.105	0.0041
7	12.020	2.053	19.576	1.2709

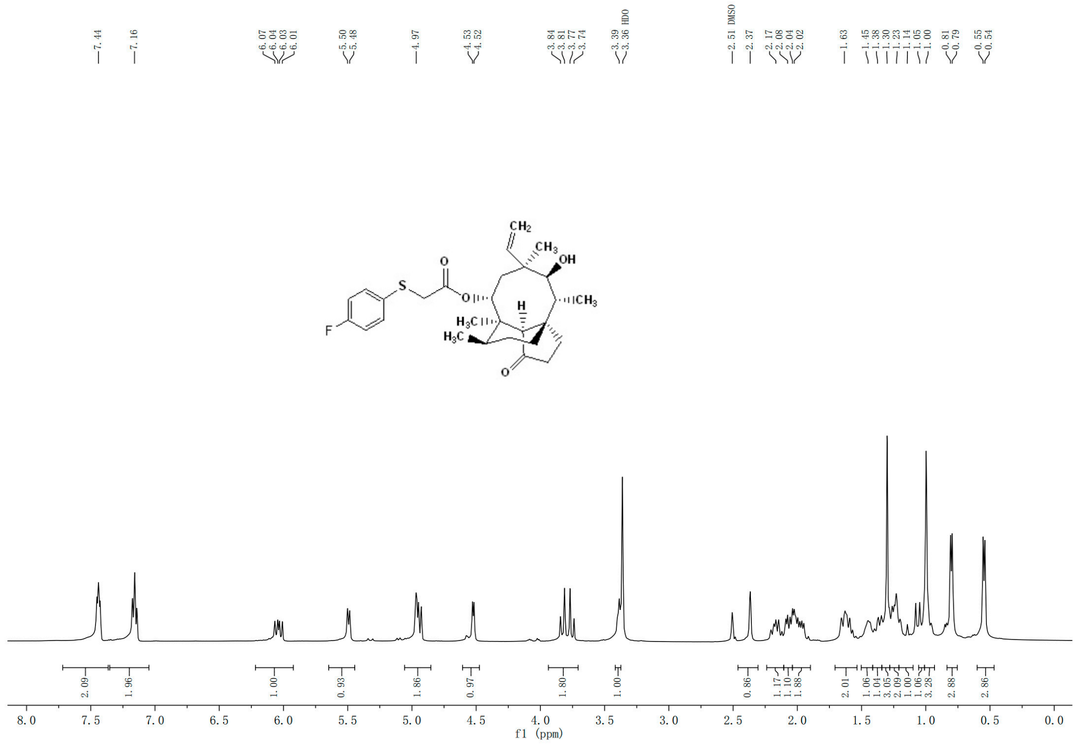

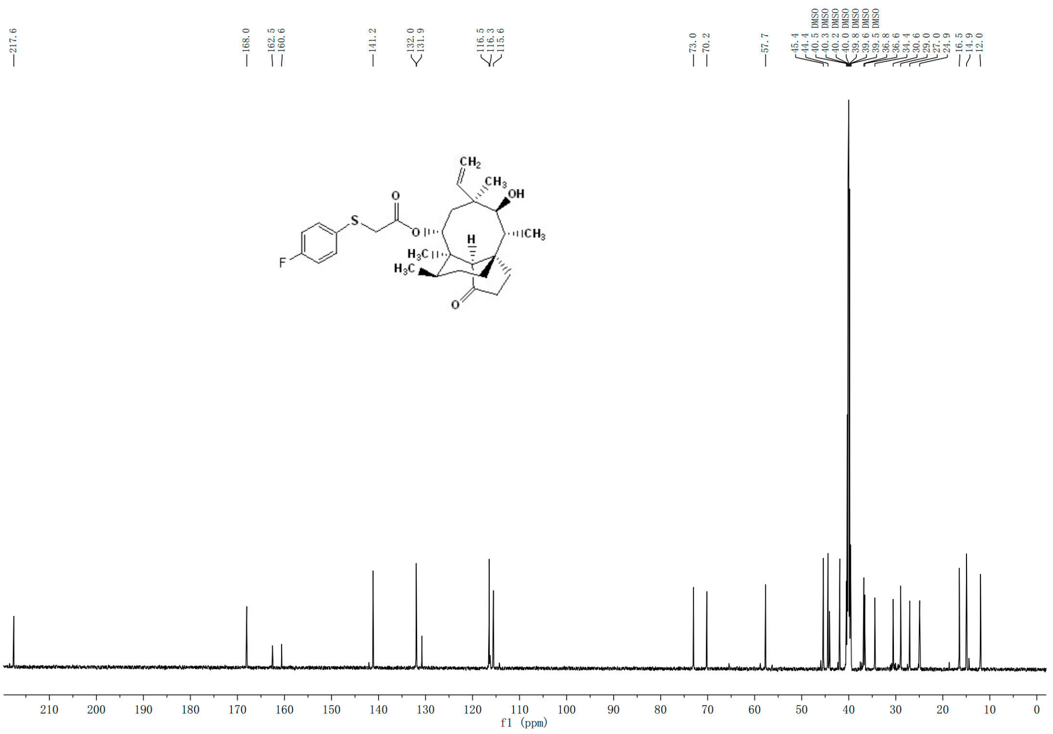

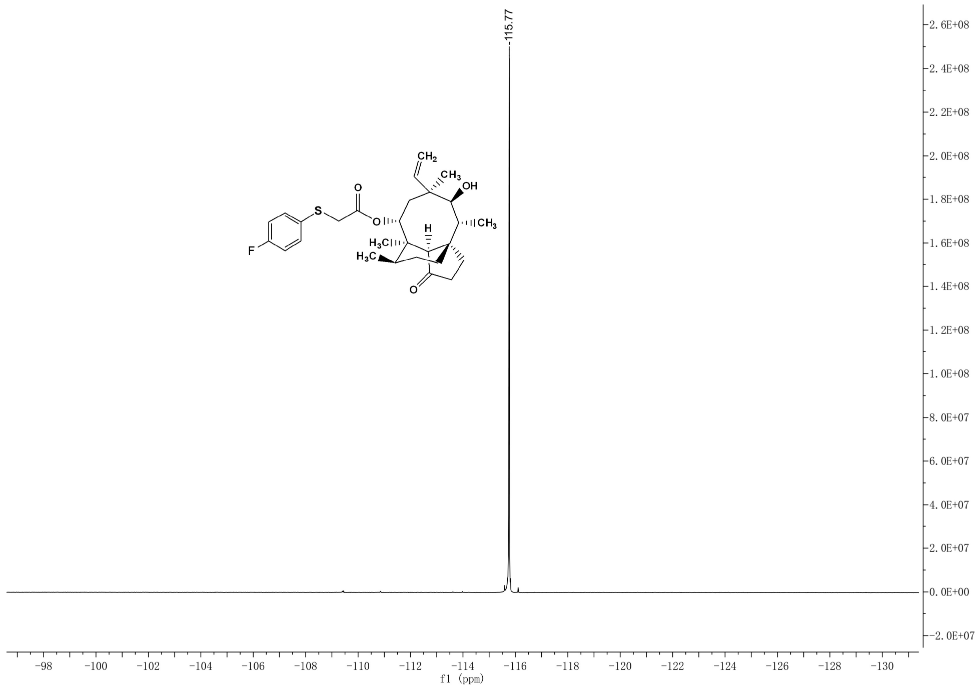

Figure S4. ^{1}H NMR, ^{13}C NMR and ^{19}F NMR spectrum of PL-W

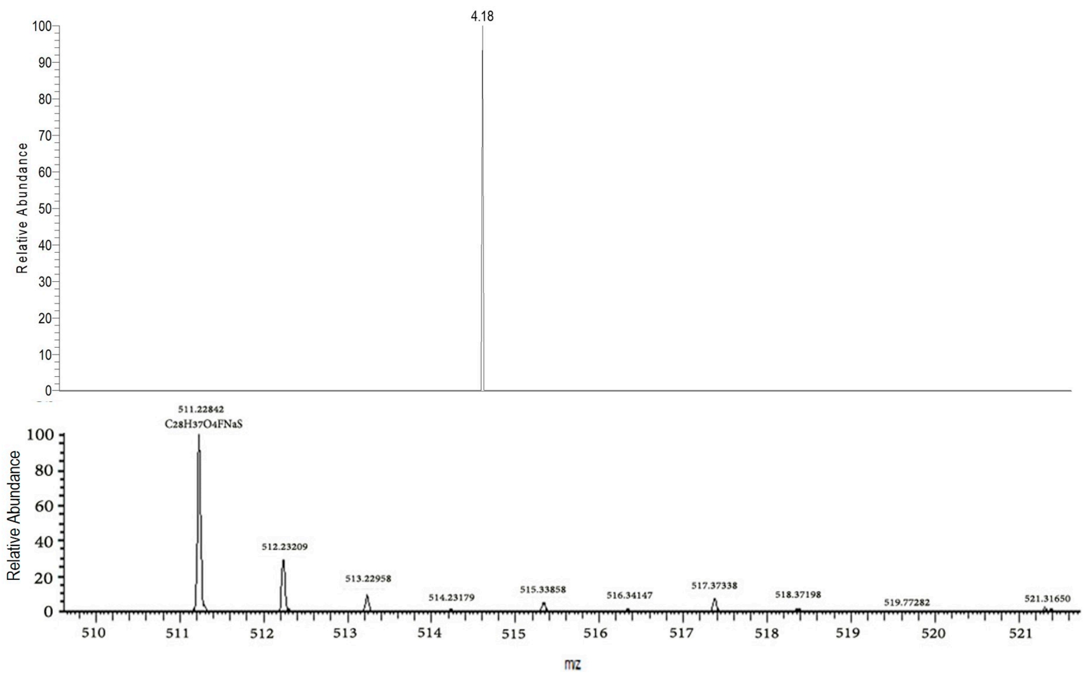

Figure S5. HRMS profile of PL-W.

Method Info: Waters XBridge C18 (150 x4.6mm, 5μm), 1.000ml/min, A: 10mmol/L NH_4HCO_3 in H_2O, B: ACN. Equilibration was carried out with a A/B solution (60.0/40.0). The flow phase ratio of A/B (60.0/40.0) was kept constant within 15 min of detection.

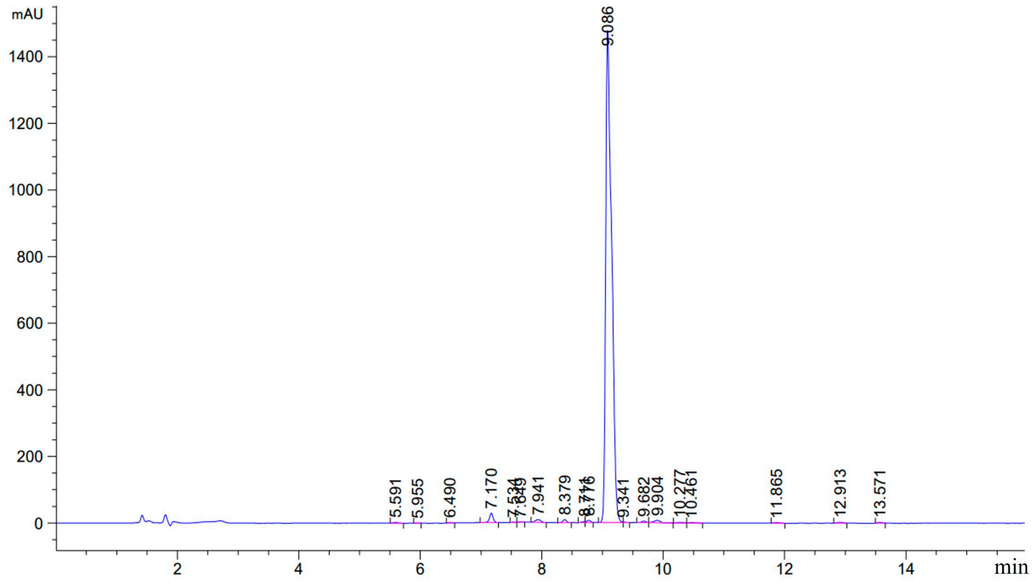

Figure S6. HPLC chromatogram of PL-W.

Table S2. HPLC integral table of PL-W

Peak	RetTime	Area(mAU*s)	Height(mAU)	**Area%**
1	5.591	9.99718	2.26562	0.0965
2	5.955	1.83496	5.10477e-1	0.0177
3	6.490	6.24570	1.63751	0.0603
4	7.170	122.08036	28.60770	1.1790
5	7.534	3.46093	1.18097	0.0334
6	7.649	2.67666	8.59818e-1	0.0258
7	7.941	50.81833	8.15692	0.4908
8	8.379	40.51814	9.42731	0.3913
9	8.711	10.63382	2.53868	0.1027
10	8.776	29.55349	6.68363	0.2854
11	9.086	9940.82617	1481.73169	96.0003
12	9.341	3.47310	1.37796	0.0335
13	9.682	22.20761	4.66831	0.2145
14	9.904	53.57173	7.39165	0.5174
15	10.277	11.63828	1.67659	0.1124
16	10.461	10.72712	1.39844	0.1036
17	11.865	8.59070	1.45163	0.0830
18	12.913	14.46059	2.47000	0.1396
19	13.571	11.67833	2.47567	0.1128

Table S3. Docking scores and intermolecular interactions and distances

Ligand	Receptor	△ Gb(kcal/mol)	Number of hy-drogen bonds	Compound atoms (functional groups)	Receptor residues	Hydrogen bond lengths
PL-W	5HL7	-9.7	2	OH(octatomic ring),<br>O(ester bond)	G2061<br>G2504	3.31<br>3.20
Tiamu-lin	5HL7	-8.7	2	OH(octatomic ring),<br>O(ester bond)	G2061<br>G2504	3.41<br>3.23
Lefamu-lin	5HL7	-9.5	2	OH(octatomic ring),<br>O(ester bond)	G2061<br>G2504	3.20<br>3.20
PL-W	1XBP	-9.0	2	OH(octatomic ring),<br>O(ester bond)	G2061<br>G2504	3.30<br>3.20
Tiamu-lin	1XBP	-8.8	2	OH(octatomic ring),<br>O(ester bond)	G2061<br>G2504	3.15<br>3.27

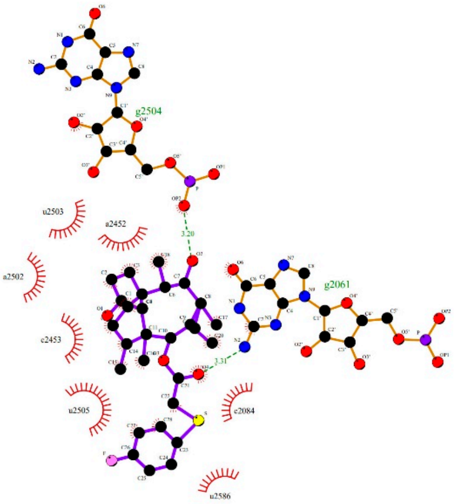

Figure S7. 2D plot of 1XBP
